# The Fate of Reoperation After Proximal Femur Fracture Surgery in Elderly Population

**DOI:** 10.7759/cureus.39856

**Published:** 2023-06-02

**Authors:** Tayfun Bacaksiz, Ihsan Akan

**Affiliations:** 1 Department of Orthopedics and Traumatology, Izmir Katip Celebi University, Izmir, TUR; 2 Department of Orthopedics and Traumatology, Izmir Katip Celebi University Ataturk Training and Research Hospital, Izmir, TUR

**Keywords:** complications, postoperative infection, reoperation, hemiarthroplasty, hip fracture

## Abstract

Introduction: The overall complication rate after proximal femur fracture surgery is high. This study aims to define the reoperation reasons and outcomes of reoperations after proximal femur fracture surgery in elderly patients.

Methods: This retrospective cohort study included patients over 75 years of age who underwent surgery for an intertrochanteric femur fracture and femoral neck fracture between 2014 and 2021. The minimum follow-up was 12 months, or until the patient was deceased. The primary outcome measure was the success of reoperation with regard to fracture type and implant.

Results: A total of 89 patients required reoperation for an overall rate of 9.3% during follow-up. Infection was the leading reason for reoperation. Hemiarthroplasty (HA) for intertrochanteric fracture is associated with a high rate of infection compared with HA for femoral neck fracture. The success rate of reoperation due to postoperative infection was poor (46.3%) whereas the success rate for other implant-related complications was favorable (91.6%).

Conclusion: The risk of postoperative infection after HA is significantly higher for intertrochanteric femur fractures compared to neck fractures in the elderly population. The limited success after postoperative infection should be taken into consideration in decision-making.

## Introduction

Proximal femoral fractures are a major health concern in the elderly population presenting with high morbidity and mortality rates. Multiple implants, such as cephalomedullary nails (proximal femoral nail [PFN]), hemiarthroplasties (HAs), and total hip arthroplasties (THAs), may be used for the surgical treatment of proximal femoral fractures. Reduced bone quality leads to implant failure and medical comorbidities lead to postoperative infections and increased mortality rates, which have been well described in the related literature [1-4]. Reoperations for implant-related infection and implant failure are challenging [5]. Despite the improvements in surgical techniques and hip implants, the reported postoperative complication rates after reoperations are considerably higher than the primary prosthetic hip procedures. Reoperation rates may be as high as 20% after surgery for hip fractures in the elderly population [6,7]. This study aims to ascertain the implant-related complications and reoperations and evaluate the success rates of reoperations after the initial operative treatment of patients with femoral neck or intertrochanteric fractures in the elderly population.

## Materials and methods

We performed a single-center retrospective analysis of a database of patients treated for proximal femoral fracture in a large training and research hospital between January 2014 and January 2021. The reoperation rate due to early postoperative joint infection (PJI) and other implant-related complications were reviewed with regard to fracture type and implant. Patients who needed reoperation for surgical complications after the index hip fracture operation were included in the study.

The inclusion criteria for hip fracture were a displaced femoral neck or intertrochanteric fracture. We classified the fractures according to the AO classification. Patients with impacted femoral neck fracture and isolated trochanter major fracture were excluded. Additionally, patients with pathological fractures, those who had received chronic suppressive antibiotic therapy, and those who underwent index surgery in another hospital were excluded. We identified 989 patients with fractures of the proximal femur who were operated on during the study periods. Nine hundred and fifty-six patients who met the inclusion criteria were included in the study. Among those patients, 572 had an intertrochanteric fracture and 384 had femoral neck fractures. In intertrochanteric fractures, proximal femur nailing was used for osteosynthesis, and bipolar uncemented hip HA or total arthroplasty was used for arthroplasty. Most of the femoral neck fractures were treated with hip arthroplasty (total or partial). The distribution of various implants regarding the type of hip fracture is presented in Table 1 in detail.

**Table 1:** Distribution of patients according to age, gender, fracture type, and surgery performed. PFN: proximal femoral nail; HA: hemiarthroplasty; THA: total hip arthroplasty; IF: internal fixation; FX: fracture; SD: standard deviation.

		Intertrochanteric FX			Neck FX			Total
		PFN	HA	THA	IF	HA	THA	
Male	n	79	115	4	5	111	4	318
	Mean age (SD)	84.70 (5.13)	85.95 (5.78)	76.25 (1.5)	76.98 (1.73)	85.2 (5.98)	80.5 (2.64)	85.22 (5.74)
Female	n	133	235	6	4	253	7	638
	Mean age (SD)	84.76 (5.20)	87.58 (6.42)	77.66 (2.50)	82.66 (2.65)	86.68 (6.15)	81.6 (5.27)	86.56 (6.15)
Total	n	212	350	10	9	364	11	956
	Mean age (SD)	84.74 (5.16)	87.06 (6.26)	77.1 (2.18)	80.77 (3.63)	86.22 (6.13)	82.09 (4.08)	86.12 (6.05)

The cohort was grouped as to whether the reoperation was carried out for postoperative deep infection (Group 1) or other reasons (Group 2). A successful treatment outcome for infection was defined as the eradication of infection as per clinical and laboratory confirmation at a minimum of one-year follow-up. Prosthetic joint infections requiring recurrent debridement after initial debridement were considered failures. A successful outcome for other reoperations (Group 2) was defined as a stable hip joint allowing weight-bearing during follow-ups or until the patient deceased without any further surgical intervention after the reoperation. Approval to use medical records was given by the local Research Ethics Committee (24.03.2022;129).

Microbiology and management of deep infection

The diagnosis of deep infection includes cases that fulfilled the criteria set out by Zimmerli et al. [8]. Indication for surgery was the clinical diagnosis of infection made based on clinical signs and laboratory parameters. The duration of the infection was less than three weeks after prosthesis implantation. All diagnoses were made using fluid or tissue samples taken at the time of debridement. Surgical washout and debridement were performed for all deep infections following hip fracture surgery. The route of administration and the duration of the antibiotic treatment were guided primarily by an infectious diseases specialist. Antibiotic therapy was individualized with intravenous administration employed for 2-6 weeks, depending on the clinical response. Modular parts of the HA were exchanged in the first debridement in all patients.

Statistical analysis

The statistical analysis was performed using Statistical Package for the Social Sciences version 15 (SPSS Inc., Chicago, IL, USA). Quantitative data were expressed as mean ± standard deviation, and count data are presented as a percentage. The chi-square test (χ^2^) was used to compare the counting data among groups. Fisher’s exact test was used for unadjusted comparisons of proportions. All data were summarized in tables during the analysis. Results were accepted as statistically significant if the P-value was <0.05.

## Results

**Figure 1:**
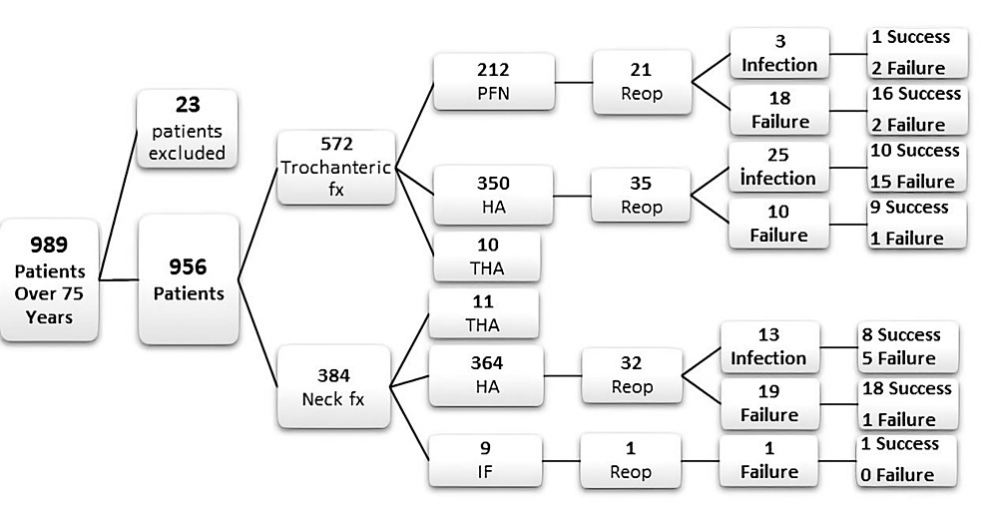
Flow chart of patients in the study PFN: proximal femoral nail; HA: hemiarthroplasty; THA: total hip arthroplasty; IF: internal fixation; FX: fracture; Reop: reoperation.

The mean age of the patients was 86.1 (75-106) years. The mean time to the first surgery for trochanteric fractures was 2.8 (1-7) days, while it was 3.1 (1-8) days for femoral neck fractures (P=0.007). A total of 89 patients required reoperation for an overall rate of 9.3%. Reasons for reoperation are summarized in Table 2. The rate of reoperations due to implant failure or infection was 9.3% (89 patients) in this series. The reasons for revision and success rates according to the type of fracture and the implants used after the operation are given in the flow chart in Figure 1. Forty-one patients needed reoperation due to a postoperative deep infection in this study (4.2%). The rate of postoperative deep infection in the HA group was 5.3%. The rate of PJI after HA for intertrochanteric fractures was 7.1%. The duration of the infection was less than three weeks after prosthesis implantation in all patients. The distribution of microorganisms revealed 61% gram-negative (GN) microorganisms (Table 3).

**Table 2:** Reason for reoperation and type of surgery performed. PFN: proximal femoral nail; HA: hemiarthroplasty; IF: internal fixation; FX: fracture.

	Trochanteric Fx		Neck Fx		Total
Reason for reoperation	PFN	HA	IF	HA	
Implant failure	18	1	0	1	20
İnfection	3	25	0	13	41
Loosening	0	2	0	4	6
instability	0	5	0	4	9
Protrusion	0	2	0	2	4
Periprosthetic fracture	0	0	0	7	7
Others (screw irritation etc.)	0	0	1	1	2
Total	21	35	1	32	89

**Table 3:** Distribution of causative microorganisms in infected cases. ITF: intertrochanteric fracture; FNF: femoral neck fracture; PFN: proximal femoral nail; HA: hemiarthroplasty.

Organism	ITF	FNF	PFN	HA	Failure	Success
*Staphylococcus*	7	5	0	12	6	6
Enterococcus faecium	5	1	0	6	3	3
Pseudomonas aeruginosa	2	2	0	4	3	1
Klebsiella pneumoniae	4	2	1	5	4	2
Acinetobacter baumannii	3	1	1	3	2	2
Polymicrobial	2	0	0	2	2	0
Enterobacter cloaca	1	0	0	1	0	1
*Escherichia coli*	2	0	1	1	1	1
Culture negative	2	2	0	4	1	3

The overall success rate of reoperation for infection was 46.3%. There was a significant difference in postoperative deep infection rate among patients in the HA group regarding the fracture type (P=0.025). However, the success rates of reoperations after infection revealed no significant difference (P=0.179).

Forty-eight patients underwent reoperation for all other reasons such as implant failure, protrusion, and pertrochanteric fractures (53.9%). The success rate of reoperations was 91.6% in this group. While two of the four cases of failure were due to infection, the other two were periprosthetic fractures. The comparison of success rates of reoperations among groups revealed a significant difference (P<0.001). Besides, the average number of operations for patients who were reoperated due to infection was 3.63, while the average number of operations for patients who were reoperated for other reasons was 1.1 (P<0.001).

Twenty-one patients in the internal fixation group underwent reoperation. Three cases had a postoperative deep infection. Among 18 patients who needed reoperation due to implant failure, one patient had undergone revision internal fixation with PFN, and the other 17 patients had a conversion to arthroplasty. Of the 18 patients, two needed reoperation due to prosthesis dislocation and periprosthetic fracture, whereas 16 patients had no complications directly related to the reoperation surgery.

## Discussion

Reoperation was performed in 89 patients (9.3%) during the follow-up period. This result is well correlated with the study of Fleekwert et al which reported a 75% complication and 11.3% reoperation rate [9]. The two most common indications for reoperation were infection and implant failure. The rate of reoperations due to implant failure is higher than the reoperation rate due to infection in the current literature [8-10]. However, most of these studies included a younger patient population. This cohort was conducted in patients over 75 years of age where HA is the most preferred treatment regardless of the hip fracture type.

The reported rates of PJI after hip arthroplasty for hip fractures vary between 0 and 18% with an average PJI rate of 2-4% [11,12]. The poor medical status of patients with older age increases associated comorbidity factors, and surgery performed for a hip fracture associated with a fracture hematoma increases the risk of PJI. Our data revealed that infection is the primary cause of reoperation after hip fracture surgery. The risk of postoperative deep infection rate is significantly higher in the HA group for intertrochanteric fractures in patients over the age of 75. This result is not well correlated to the current literature. Kumar et al. in a meta-analysis reported a comparable and more importantly small risk of postoperative infection in the treatment of intertrochanteric fractures via internal fixation or HA [13]. However, Jolly A. recently reported an 8% PJI rate after bipolar HA for intertrochanteric fractures [14].

HA for femoral neck fracture is relatively a less extensive procedure compared with HA for intertrochanteric fractures. Considering the nature of hip surgery, larger preoperative hematoma (extracapsular fracture) and larger bone excision may predispose a higher risk of PJI after HA for intertrochanteric fractures when compared to HA for femoral neck fractures.

There is no consensus regarding the treatment of intertrochanteric fractures in the elderly and the most appropriate treatment is still not clear. The attractive traditional treatment modality is internal fixation via PFN [15]. On the other hand, PFN has been associated with many mechanical complications and high failure rates, especially in unstable osteoporotic fracture patterns [14,16,17]. We found a reoperation rate of 9.9%, which is very close when compared to the HA group (10%). However, the prognosis of conversion of the failed internal fixation to HA or THA is favorable in our study whereas the management of postoperative PJI after HA is not. These results are consistent with the relative literature [12]. 

The traditional management of early (acute) onset PJI after arthroplasty is DAIR (debridement, antibiotics, irrigation, and retention) treatment. However, the reported success rates of DAIR treatment after hip HA procedures are conflicting with variable success rates [13,18,19]. Our results revealed that the success rate of DAIR treatment after HA is limited regardless of the fracture type. The effectiveness of DAIR treatment, comparing primary THA infections with HA infections in terms of success rate and epidemiological factors, reveals inferior results in the literature. This may be attributed to the nature of surgery [17]. Del Toro et al. stated that tissue damage in hip fracture patients favors the devolvement of infection and also jeopardizes surgical treatment [20].

We found a higher rate of GN infections in these series which may have clinical importance because treatment of such infections is considered more complicated by the virulence of the organisms and their growing resistance to most available antibiotics. Relevant studies revealed that GN infections tend to occur more frequently in elderly patients and in the early postoperative period as an important factor contributing to the poor outcome of DAIR treatment [20,21]. Frequently associated with no control of rectal and urinary sphincter mechanisms are sources of contamination with GN bacterium and *Enterococcus* species in these fragile patient populations [13].

The study has some limitations. It is a single-center retrospective study. However, the patient number is comparatively large, making this a relevant sample of Turkey hip fracture patients. Reoperation comprises a wide range of different events, which makes a detailed analysis impossible where multiple surgical implants are used based on the surgeon’s preferences and fracture characteristics.

## Conclusions

This study was conducted in patients over 75 years of age where HA is the most preferred treatment regardless of the hip fracture type. In conclusion, this retrospective cohort study indicates that the risk of infection after HA is significantly increased in hip fracture surgery and that reoperation due to infection is less successful than other causes. This is more noticeable in HA patients who were performed on this trochanteric fracture site.

## References

[1] Yam M, Kang BJ, Chawla A (2020). Cephalomedullary blade cut-ins: a poorly understood phenomenon. Arch Orthop Trauma Surg.

[2] Brazilian Society of Orthopedics and Traumatology, Brazilian College of Radiology (2011). Transtrochanteric fractures. Rev Assoc Med Bras (1992).

[3] Park MS, Cho HM, Kim JH, Shin WJ (2013). Cementless bipolar hemiarthroplasty using a rectangular cross-section stem for unstable intertrochanteric fractures. Hip Int.

[4] Fitzpatrick P, Kirke PN, Daly L (2001). Predictors of first hip fracture and mortality post fracture in older women. Ir J Med Sci.

[5] Appelt A, Suhm N, Baier M, Meeder PJ (2007). Complications after intramedullary stabilization of proximal femur fractures: a retrospective analysis of 178 patients. Eur J Trauma Emerg Surg.

[6] Kazimoglu C, Yalcin N, Onvural B, Akcay S, Agus H (2015). Debridement, antibiotics, irrigation, and retention (DAIR) of the prosthesis after hip hemiarthroplasty infections. Does it work?. Int J Artif Organs.

[7] Tiihonen R, Alaranta R, Helkamaa T, Nurmi-Lüthje I, Kaukonen JP, Lüthje P (2019). A 10-year retrospective study of 490 hip fracture patients: reoperations, direct medical costs, and survival. Scand J Surg.

[8] Zimmerli W, Trampuz A, Ochsner PE (2004). Prosthetic-joint infections. N Engl J Med..

[9] Flikweert ER, Wendt KW, Diercks RL, Izaks GJ, Landsheer D, Stevens M, Reininga IH (2018). Complications after hip fracture surgery: are they preventable?. Eur J Trauma Emerg Surg.

[10] Viberg B, Pedersen AB, Kjærsgaard A, Lauritsen J, Overgaard S (2022). Risk of mortality and reoperation in hip fracture patients undergoing cemented versus uncemented hemiarthroplasty: a population-based study from Danish National Registries. Bone Joint J.

[11] Taheriazam A, Saeidinia A (2017). Conversion of failed hemiarthroplasty to total hip arthroplasty: a short-term follow-up study. Medicine (Baltimore).

[12] Yassin M, Sharma V, Butt F, Iyer S, Tayton E (2020). Early peri-prosthetic joint infection after hemiarthroplasty for hip fracture: outcomes of debridement, antibiotics, and implant retention. Surg Infect (Larchmt).

[13] Kumar P, Rajnish RK, Sharma S, Dhillon MS (2020). Proximal femoral nailing is superior to hemiarthroplasty in AO/OTA A2 and A3 intertrochanteric femur fractures in the elderly: a systematic literature review and meta-analysis. Int Orthop.

[14] Jolly A, Bansal R, More AR, Pagadala MB (2019). Comparison of complications and functional results of unstable intertrochanteric fractures of femur treated with proximal femur nails and cemented hemiarthroplasty. J Clin Orthop Trauma.

[15] Lora-Tamayo J, Euba G, Ribera A (2013). Infected hip hemiarthroplasties and total hip arthroplasties: differential findings and prognosis. J Infect.

[16] Verzellotti S, Candrian C, Molina M, Filardo G, Alberio R, Grassi FA (2020). Direct anterior versus posterolateral approach for bipolar hip hemiarthroplasty in femoral neck fractures: a prospective randomised study. Hip Int.

[17] Hoffmann M, Hartel M, Rueger JM, Lehmann W (2014). Primary prosthetic replacement in per- and intertrochanteric fractures. Eur J Trauma Emerg Surg.

[18] Özkayın N, Okçu G, Aktuğlu K (2015). Intertrochanteric femur fractures in the elderly treated with either proximal femur nailing or hemiarthroplasty: A prospective randomised clinical study. Injury.

[19] de Jong L, Klem TM, Kuijper TM, Roukema GR (2017). Factors affecting the rate of surgical site infection in patients after hemiarthroplasty of the hip following a fracture of the neck of the femur. Bone Joint J.

[20] del Toro MD, Nieto I, Guerrero F (2014). Are hip hemiarthroplasty and total hip arthroplasty infections different entities? The importance of hip fractures. Eur J Clin Microbiol Infect Dis.

[21] Hsieh PH, Lee MS, Hsu KY, Chang YH, Shih HN, Ueng SW (2009). Gram-negative prosthetic joint infections: risk factors and outcome of treatment. Clin Infect Dis.

